# Efficacy of Chemotherapies and Stem Cell Transplantation for Systemic AL Amyloidosis: A Network Meta-Analysis

**DOI:** 10.3389/fphar.2019.01601

**Published:** 2020-01-28

**Authors:** Yuwen Cai, Shizhang Xu, Na Li, Song Li, Gaosi Xu

**Affiliations:** ^1^ Department of Nephrology, the Second Affiliated Hospital of Nanchang University, Jiangxi, China; ^2^ Second Clinical Medical College of Nanchang University, Jiangxi, China; ^3^ Department of Nephrology, People’s Hospital of Yichun City, Yichun, China; ^4^ The School of Nursing, Nanchang University, Jiangxi, China

**Keywords:** amyloidosis, chemotherapy, outcomes research, autologous stem cell transplantation, network meta-analysis

## Abstract

**Background/Aims:**

The present Bayesian network meta-analysis (NMA) was to compare the efficacy of different chemotherapies and autologous stem cell transplantation (ASCT) in immunoglobulin light-chain (AL) amyloidosis.

**Methods:**

We systematically searched PubMed, Embase, Web of Science, and the Cochrane Central Register of Controlled Trials (CENTRAL) for studies compared the rates of hematological response (HR), complete response (CR), renal response, and cardiac response in AL amyloidosis patients.

**Results:**

There were three randomized controlled trials (RCTs) and thirteen observational controlled trials (OCTs) comprising 3,402 participants enrolled for the comparisons of seven treatments: melphalan + dexamethasone (MDex), high-dose melphalan followed by ASCT, bortezomib + melphalan + dexamethasone (BMDex), thalidomide + cyclophosphamide + dexamethasone (CTD), bortezomib + dexamethasone (BDex), bortezomib + cyclophosphamide + dexamethasone (CyBorD), cyclophosphamide + lenalidomide + dexamethasone (CLD). BMDex was ranked first in the aspect of both HR and CR, CTD induced the highest rate of renal response, and BDex was possibly the best treatment for the cardiac response.

**Conclusion:**

Although more data about safety and cost are needed, BMDex was recommended as the most efficient treatment for AL amyloidosis patients for enhancing the response rate for HR and CR.

## Introduction

Systemic immunoglobulin light chain (AL) amyloidosis is the most common type of systemic amyloidosis. It is a life-threatening disease related to monoclonal light chains, which are produced by clonal plasma cells in the bone marrow and can deposit both in vital organs and systemically (Comenzo et al., 2012). It has an estimated incidence rate of 8 to 10 cases per million person-years, and the deposition of monoclonal light chains in vital organs, especially the kidney, heart, liver, soft tissue, and nerves, can cause progressive organ dysfunction and death (Kyle et al., 1992; Merlini and Palladini, 2008). Therefore, it is very important to study the effective treatment for this disease.

Various therapies have been investigated in AL amyloidosis patients, which includes alkylating agents like melphalan and cyclophosphamide, immunomodulatory drugs like thalidomide, lenalidomide, proteasome inhibitor bortezomib, and autologous stem cell transplantation (ASCT) (Gatt and Palladini, 2013; Palladini and Merlini, 2016). These agents can be used alone or in combination with each other. Unfortunately, the comparative of these therapies remains unproven.

A traditional pairwise meta-analysis performed in 2009 (Mhaskar et al., 2009) led to a conclusion that ASCT does not appear to be superior to conventional chemotherapy. However, a retrospective study with a large sample size in 2018 (Shimazaki et al., 2018) still found a higher response rate in patients treated with ASCT than in patients treated with melphalan and dexamethasone (MDex). In addition, traditional pairwise meta-analyses were not able to synthesize all evidence simultaneously and rank the treatments since studies comparing treatments directly were lacking (Lumley, 2002; Caldwell et al., 2005). Therefore, it is important to perform this network meta-analysis (NMA), which was able to test the firmness of the pairwise meta-analyses and supply missing data for direct comparisons by combining direct and indirect evidence, to explore the efficacy of various therapies in systemic AL amyloidosis.

## Methods

### Data Sources and Search Strategy

This study was conducted according to the Preferred Reporting Items for Systematic Reviews incorporating Network Meta Analyses (PRISMA-NMA) statement (Hutton et al., 2015). We systematically searched EMBASE, PubMed, Web of Science, and the Cochrane Central Register of Controlled Trials (CENTRAL) for articles from January 2005 (this was when the 10th International Symposium on Amyloid and Amyloidosis suggested the criteria for organ involvement and response) (Gertz et al., 2005) up to January 2019. No language restrictions were set during the searches. The search strategy was as follows: [(Immunoglobulin light-chain Amyloidosis) or (Immunoglobulin light-chain Amyloidoses) or (AL Amyloidosis) or (AL Amyloidoses) or (Primary Systemic Amyloidosis) or (Primary Systemic Amyloidoses)] and [(Drug therapy) or (Drug Therapies) or (Chemotherapy) or (Chemotherapies) or (Pharmacotherapy) or (Pharmacotherapies) or (Stem Cell Transplantation)]. We also reviewed relevant research references for additional trials. The last search date was May 1, 2019.

### Selection Criteria

We collected all randomized controlled trials (RCTs) and observational controlled trials (OCTs) comparing the efficacy of different treatments in patients with AL Amyloidosis. Enrolled studies satisfied the following requirements: (1) patients were at least eighteen year old and had biopsy-proven systemic AL amyloidosis, (2) the information of interventions included ASCT, MDex, bortezomib in combination with dexamethasone (BDex), bortezomib in combination with melphalan and dexamethasone (BMDex), bortezomib in combination with cyclophosphamide and dexamethasone (CyBorD), cyclophosphamide in combination with thalidomide and dexamethasone (CTD), or cyclophosphamide in combination with lenalidomide and dexamethasone (CLD), (3) articles provided exact data of hematological response (HR), complete response (CR), renal response or cardiac response among patients receiving different treatments, and (4) study design was RCTs or OCTs. The detailed selection criteria were shown in **Supplement 1**, and the exclusion criteria were as follows: (1) studies were case reports, reviews, editorials or comments, etc., (2) the subjects with familial, cutaneous or transthyretin-related amyloidosis, or with relapsed AL amyloidosis, and (3) the type of treatment was not described clearly or was not included in our study.

### Data Extraction and Quality Evaluation

Two authors (YC and SX) extracted the data from the eligible articles independently using standard data collection sheets. Information collected from the enrolled studies included the first author’s name, year of publication, country and language information, mean time of follow-up, study design, number of participants, age of patients, type of interventions, organ involvements, and clinical outcomes. We used version 2 of the Cochrane risk-of-bias tool for randomized trials (RoB 2) to evaluate the quality of enrolled RCTs (Sterne et al., 2010) and Newcastle-Ottawa scale (NOS) for OCTs (Stang, 2010). RoB 2 has five domains for enrolled RCTs: bias arising from the randomization process, bias due to deviations from intended interventions, bias due to missing outcome data, bias in measurement of the outcome, bias in selection of the reported result. And each domain of each study was classified into: high risk of bias, some concerns or low risk of bias. For enrolled OCTs, the quality was scored according to three items: selection, comparability, and outcome. Studies with 7 to 9 total scores are of high quality, while those with 4 to 6 total scores are of medium quality. Disputes were resolved through discussion, or a third author (Gaosi Xu) would decide.

### Statistical Analysis

The rates of HR, CR, renal response and cardiac response were collected from the enrolled studies. Then the relative efficacy was measured using odds ratios (ORs) and their corresponding 95% confidence intervals (CIs). We used STATA (version 14.0, Stata MP) as well as R software (version 3.5.1) to abstract and analyze the data.

Traditional pairwise meta-analyses were first performed to combine studies reporting the same clinical outcomes and studied therapies, then a network meta-analysis considering multiple therapies was performed in a Bayesian random-effect model using the “gemtc” R package. It was done by recalling JAGS in R for Markov chain Monte Carlo (MCMC) sampling. For each analysis, we generated 200,000 simulations for each of the sets of different initial values and discarded the first 5,000 simulations as the burn-in period. Then we checked the convergence using Brooks-Gelman-Rubin diagnostic and trace plots (Gelman, 1998).

The robustness of each analysis was assessed by sensitivity analyses with omitting each study sequentially. Additionally, ranking probabilities for the treatments were obtained using the surface under the cumulative ranking area (SUCRA) (Salanti et al., 2011). Inconsistency between direct and indirect calculations was tested using the node-splitting method. The method separated direct and indirect evidence concerning the same comparisons, and reported the inconsistency using *P* value (Spiegelhalter et al., 2002). Heterogeneity was evaluated using *I* ^2^ test, and an *I* ^2^ more than 50% indicated the existence of significant heterogeneity. Considering that there were studies of different designs enrolled in the analysis, we also used the design-adjusted model, where studies of non-RCT designs are down-weighted to account for their higher risk of bias, to examine the firmness of our results. The weighting factor ranges between 0 (exclude completely) and 1 (weight equally). We set 0.5 as the weighting factor for OCTs. This was done using a variance inflation; we replaced the likelihood “Y_ik_ ~ N (θ_ik_, S^2^_ik_)” by an inflated version “Y_ik_ ~ N (θ_ik_, S^2^_ik_/αi)”, and αi = 0.5 (Mengersen, 2004; Efthimiou et al., 2017).

## Results

### Baseline Characteristics of Enrolled Studies

**Figure 1:**
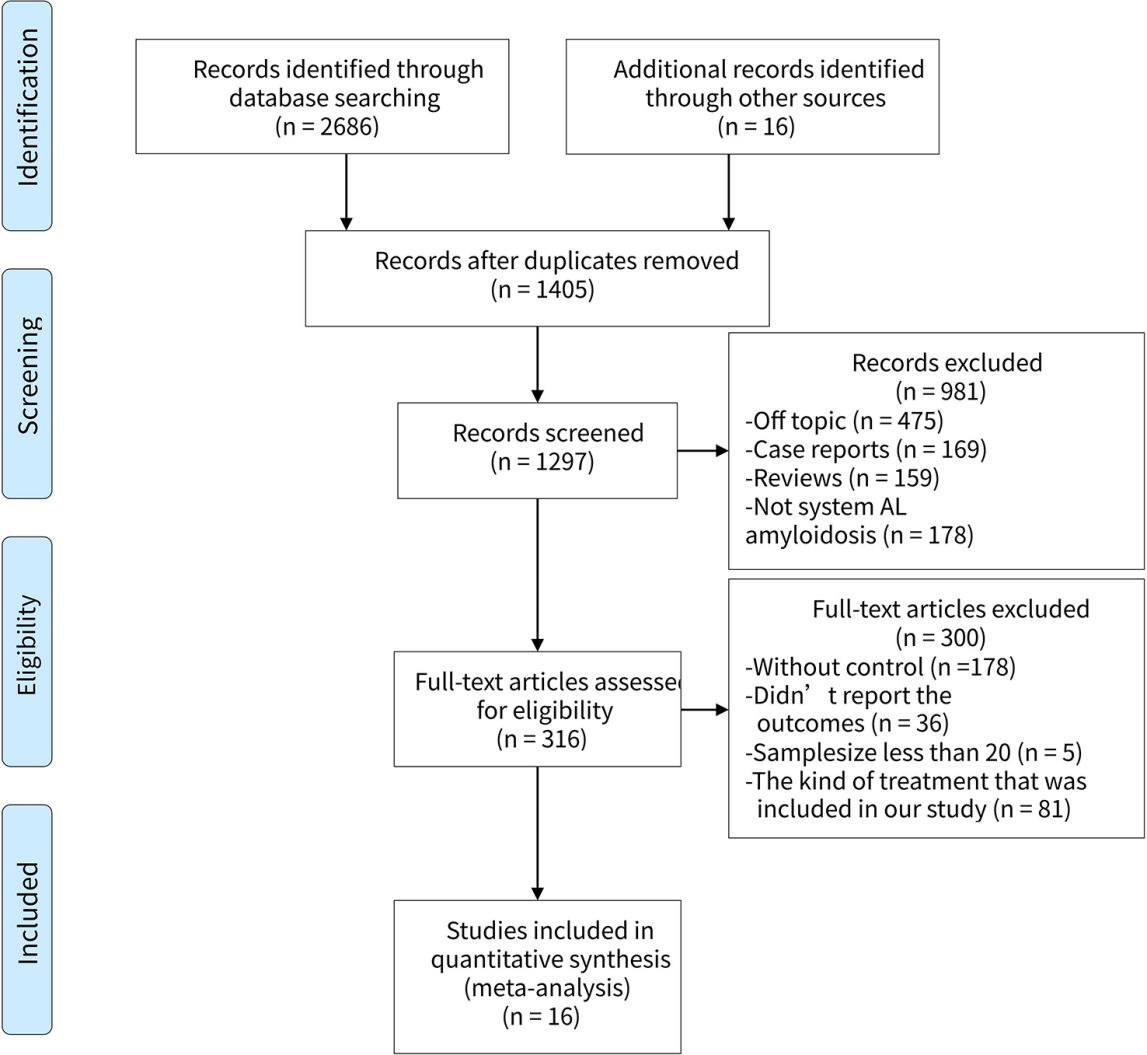
Flowchart for selection of articles to be included in the NMA.

We filtered a total of 1,406 articles, from which sixteen studies (Jaccard et al., 2008; Gibbs et al., 2009; Gillmore et al., 2009; Wechalekar et al., 2010; Wechalekar et al., 2013; Palladini et al., 2014; Venner et al., 2014; Kastritis et al., 2015; Milani et al., 2015; Gertz et al., 2016; Katoh et al., 2016; Kastritis et al., 2016; Milani et al., 2017; Kastritis et al., 2017; Shimazaki et al., 2018; Liu et al., 2019) involving 3,402 participants were ultimately selected for the analysis. Of the sixteen studies, three were RCTs (Gillmore et al., 2009; Kastritis et al., 2016), two were prospective types (Wechalekar et al., 2010; Palladini et al., 2014), the other two were case-control types (Palladini et al., 2014; Milani et al., 2015), and the remaining nine were retrospective types (Gibbs et al., 2009; Wechalekar et al., 2010; Wechalekar et al., 2013; Venner et al., 2014; Kastritis et al., 2015; Katoh et al., 2016; Kastritis et al., 2017; Shimazaki et al., 2018; Liu et al., 2019). The PRISMA flow diagram was presented in **Figure 1**. In general, the quality of these studies were medium to high, as shown in **Table 1** and **Supplement 2**. The articles enrolled were published from 2007 to 2018. **Table 2** presents the essential baseline characteristics of the articles. Of the sixteen studies, all studies submitted data on HR, twelve studies presented data on CR, nine studies provided data on renal response, and eight studies supplied data on cardiac response. The sample size of each study ranged from 24 to 796, the mean time of follow-up ranged from one to five years.

**Table 1:** Quality evaluation of enrolled OCTs according to Newcastle-Ottawa Scale (NOS).

Included study	Selection	Comparability	Outcome	Total score
Gertz et al., 2016	4	1	3	8
Liu et al., 2019	3	2	3	8
Venner et al., 2014	3	2	2	7
Kastritis et al., 2017	3	2	3	8
Kastritis et al., 2015	2	2	3	7
Milani et al., 2017	3	1	2	6
Wechalekar et al., 2010	3	1	2	6
Palladini et al., 2014	3	2	3	8
Simon DJ 2009	3	2	3	8
Shimazaki et al., 2018	4	2	3	9
Katoh et al., 2016	3	1	3	7
Wechalekar et al., 2013	4	1	3	8
Milani et al., 2015	3	1	3	7

**Table 2:** The baseline characteristics of the included studies.

Study	Country	Language	Study design	Median follow-up (m)	Interventions	Sample size	Age (y)	Male (%)	Kidney involved (%)	Cardiac involved (%)	Endpoints
Kastritis et al., 2016	Australia, Europe	English	Randomized	25	MDex, BMDex	110	59~72	56	65	78	①③④
Jaccard et al., 2008	France	French	Randomized	36	MDex, ASCT	100	49~66	57	69	47	①②③④
Gillmore et al., 2009	Britain	English	Randomized	7	MDex, CTD	24	42~ 85	NR	NR	NR	①②
Gertz et al, 2016	Britain	English	Prospective	36	MDex, ASCT	72	44~74	78	75	31	①②③④
Milani et al., 2017	Italy	English	Prospective	42	MDex,CyBorD, BMDex	796	NR	NR	66	77	①
Katoh et al., 2016	Japan	Japanese	Retrospective	12	BDex, ASCT	50	44~77	60	70	44	①②③④
Liu et al., 2019	China	Chinese	Retrospective	22	CTD, MDex	68	45~67	50	100	65	①②③④
Chihiro 2018	Japan	Japanese	Retrospective	24	MDex, ASCT	741	31~93	62	73	34	①③④
Venner et al., 2014	Britain	English	Retrospective	26	CTD, CyBorD	138	39~83	60	78	79	①②③④
Kastritis et al., 2017	Greece	English	Retrospective	36	BDex, CyBorD	101	NR	NR	71	70	①②③④
Wechalekar et al., 2013	Britain, Italy, Germany	English	Retrospective	24	MDex, CTD	346	37~88	NR	62	NR	①②③④
Efstathios 2015	Greece	English	Retrospective	57	BDex, CLD	85	42~82	57	70	67	①②
Milani et al., 2015	Italy	English	Case-control	36	BMDex, CyBorD	174	NR	NR	100	100	①③④
Palladini et al., 2014	Italy	English	Case-control	61	MDex, BMDex	174	62~74	NR	63	85	①②③④
Simon 2009	Britain	English	Retrospective	12	MDex, CTD	180	64~72	65	80	86	①②③
Wechalekar et al., 2010	Britain, Italy, Greece	English	Retrospective	29	BDex,MDex, CLD,CTD,ASCT	243	NR	NR	76	60	①②

NR, not report; ①: hematological response; ②: complete response; ③: renal response; ④: cardiac response; m, month; y, year.

### Network Structure, Consistency, and Heterogeneity

**Figure 2:**
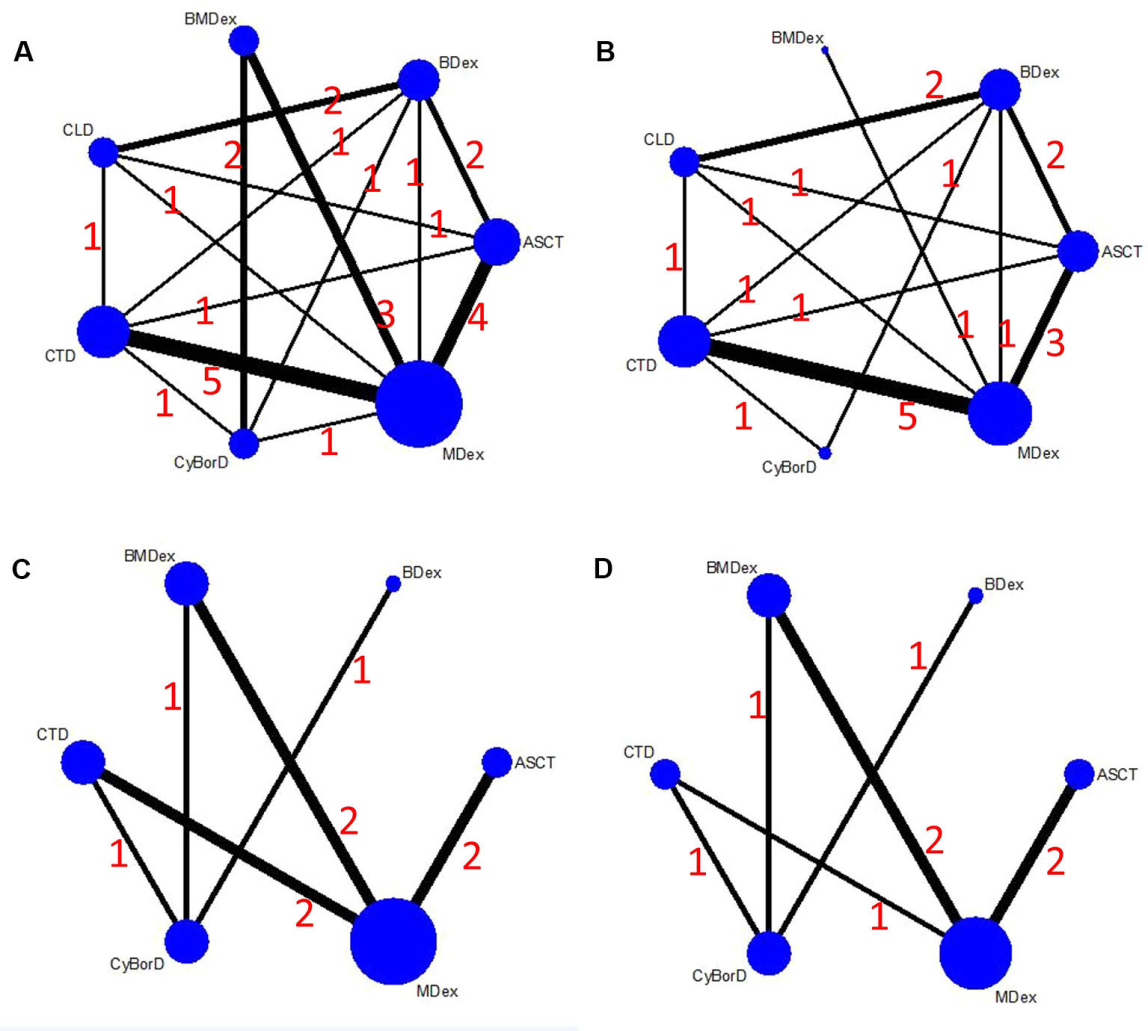
Network plots of the comparisons between different therapies: **(A)** hematological response; **(B)** complete response; **(C)** renal response; **(D)** cardiac response.

**Figure 2** shows a network plot of the efficacy comparisons for this NMA. There are six interventions for renal response and cardiac response, seven interventions for HR and CR. The node size is in proportion to the treatment’s total sample size from all enrolled studies, and the line’s thickness between the two directly compared treatments is in proportion to the number of studies. As illustrated in the network plot, the number of treatments differed among various outcomes.

The convergence of this NMA was generally satisfactory, as illustrated in the diagnostic and trace plots (**Supplement 3**). In the pairwise meta-analyses, significant heterogeneity was found in the comparisons of MDex and ASCT for HR and CR (*I* ^2^^=^ 79.0% and *I* ^2^ = 53.5%, respectively, as shown in **Supplement 4**), and that was why we chose a random-effect model. A significant difference was observed when we compared BDex and ASCT for their efficacy in HR. Compared with ASCT, OR from direct evidence indicated a higher response rate of BDex, whereas OR from indirect evidence reported a lower response rate (*P*-value was 0.02, as shown in **Supplement 5**). Nevertheless, no other statistically significant difference was found between direct and indirect evidence.

### The Efficacy Outcomes

#### HR

**Figure 3:**
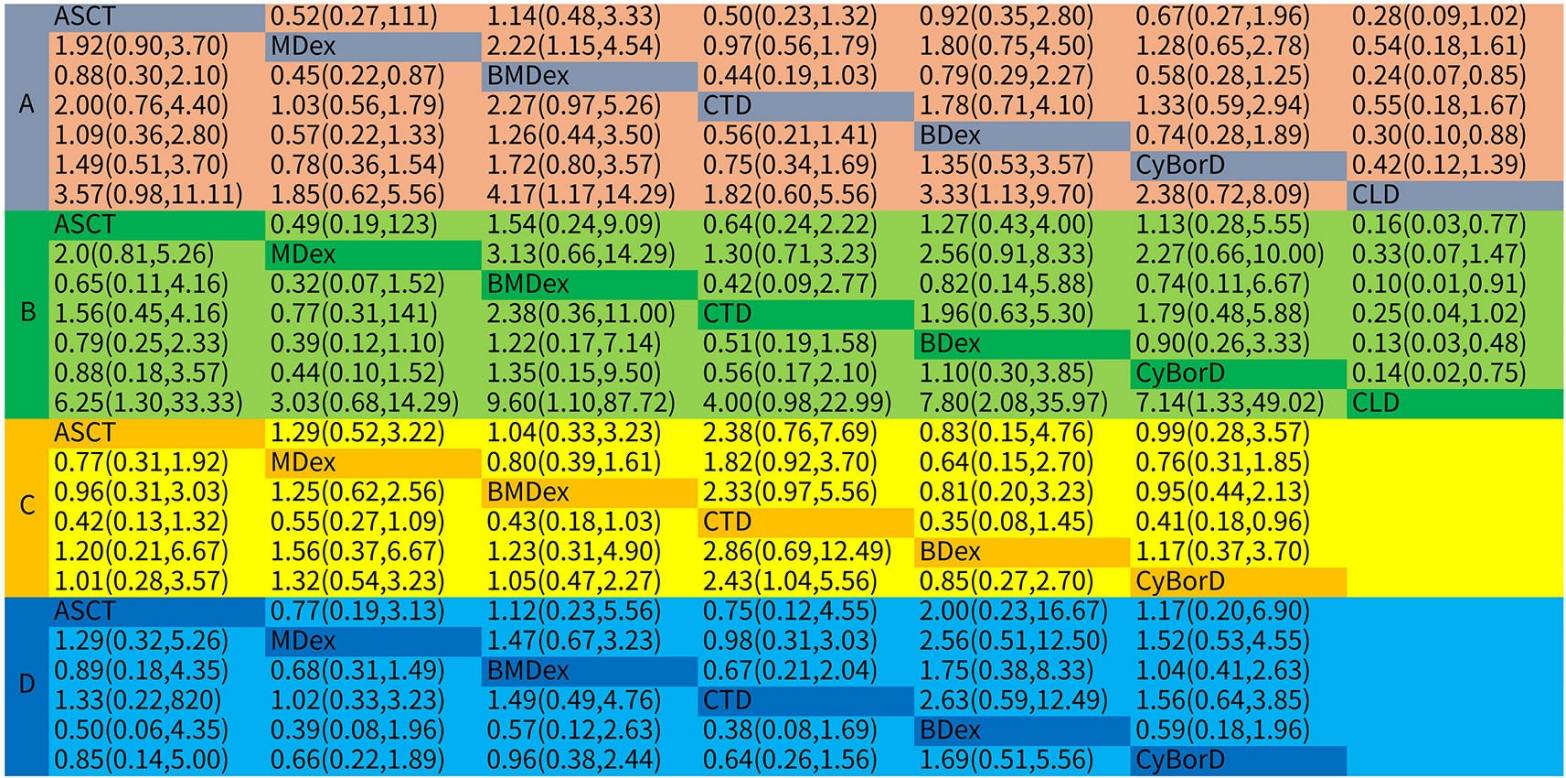
Odds ratios (ORs) with 95% confidence intervals (CrIs) for network meta-analysis comparisons over treatments: **(A)** hematological response; **(B)** complete response; **(C)** renal response; **(D)** cardiac response.

As the primary outcome of this analysis, a NMA was performed to explore the efficacy of the treatments for HR. All of the 16 studies with both direct and indirect comparisons were involved. Treatments were compared with each other separately; ORs and their corresponding 95% CrIs were considered. As demonstrated in **Figure 3**, compared with MDex, BMDex has significantly higher HR rate (OR = 2.22, 95% CrI 1.15~4.54); however, no significant difference among other treatments was observed in this NMA.

**Figure 4:**
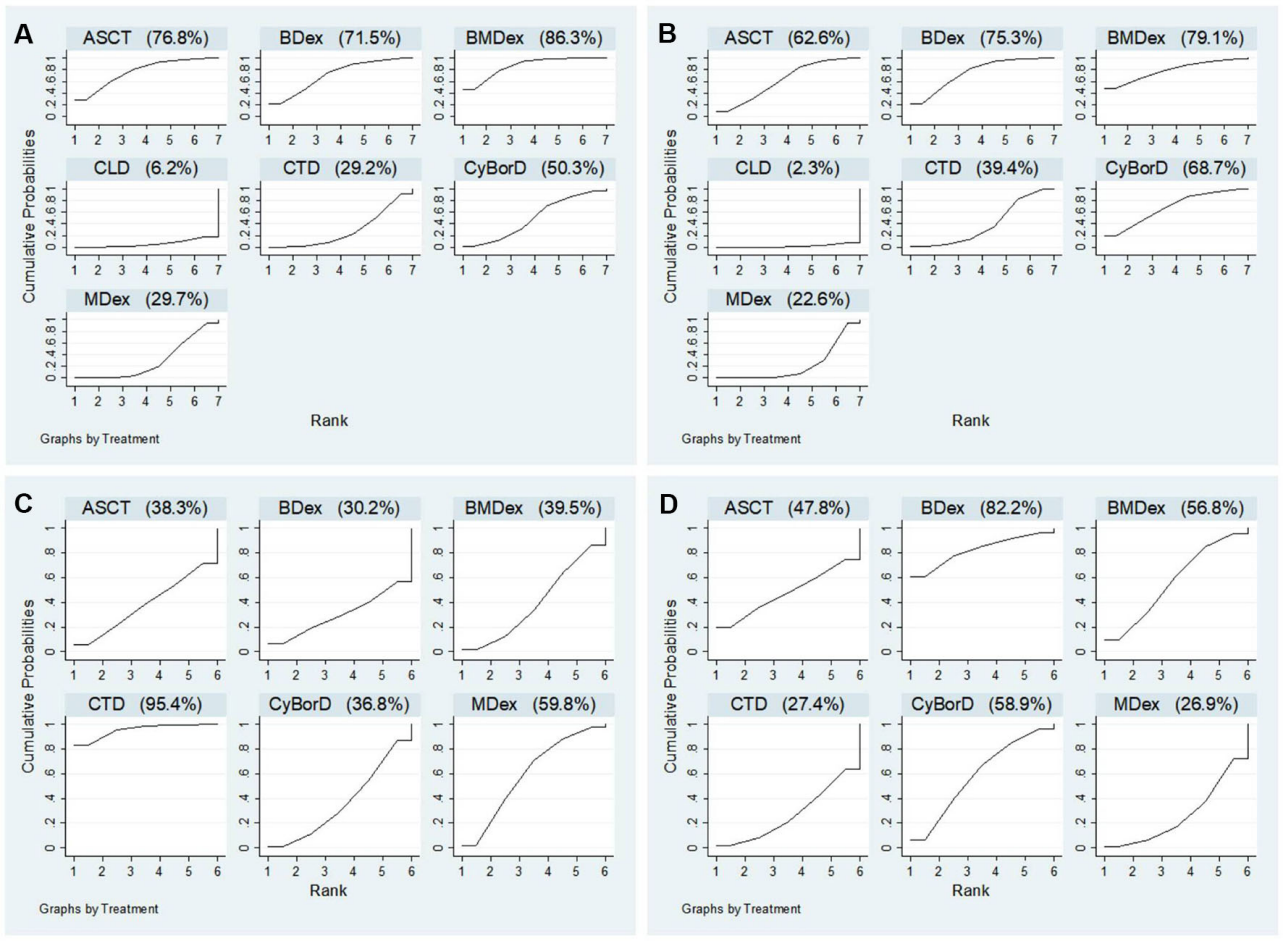
Ranking probabilities of surface under the cumulative ranking curve (SUCRA): **(A)** hematological response; **(B)** complete response; **(C)** renal response; **(D)** cardiac response.

**Figure 4** shows the comparative efficacy of treatments with SUCRA probabilities. Of all the seven studied treatments, BMDex and ASCT were ranked first and second (SUCRA of 86.3 and 76.8, respectively), followed by BDex (SUCRA of 71.5). CLD was ranked last in terms of achieving HR.

#### CR

Twelve studies were enrolled in the calculation for the efficacy of treatments on CR. We observed that ASCT, BDex, BMDex, and CyBorD were associated with increased rates of CR (OR = 6.25, CrI 1.30~33.33; 7.80, CrI 2.08~35.97; 9.60, CrI 1.10~87.72; and 7.14, CrI 1.33~49.02, **Figure 3**) compared with CLD. BMDex was most likely to be ranked first in terms of CR (SUCRA of 79.1), followed by BDex (SUCRA of 75.3), CyBorD (SUCRA of 68.7), and ASCT (SUCRA of 62.6). Similarly, CLD ranked the least effective therapy in achieving CR.

#### Renal Response

Nine studies involving six treatments were enrolled in the calculation about the renal response rate. As demonstrated in **Figure 3**, CTD has a significantly higher renal response rate than CyBorD (OR = 2.43, 95% CrI 1.04~5.56). Different from the hematological and CR, CTD was found to have the highest probability of being ranked first in the aspect of renal response (SUCRA of 95.4), followed by MDex.

(SUCRA of 59.8), whereas ASCT and bortezomib combinations were quite possible to be lower-ranking in achieving renal response, according to SUCRA.

#### Cardiac Response

Eight studies reported the outcome of the cardiac response. However, we did not get much significant results from the calculation. As shown in **Figure 4**, BDex was most likely to be ranked first (SUCRA of 82.2), followed by CyBorD and BMDex (SUCRA of 58.9 and 56.8).

### Sensitivity Analysis, Publication Bias, and Design-Adjusted Analysis

We conducted a sensitivity analysis by omitting single study sequentially, and there was no significant difference between the primary results. As shown in **Supplement 6**, No significant publication bias was detected in the funnel plot. Results of the design-adjusted analysis were also in line with the primary results.

## Discussion

In this NMA, we systematically analyzed the response characteristics of seven treatments for AL amyloidosis. Three thousand four hundred and two participants from 16 studies were involved. As illustrated in our results, BMDex was considered as the most efficient therapy for AL amyloidosis, namely the highest rates of HR and CR. CTD was regarded to be a quite efficient therapy for renal involved patients, because it induced the highest renal response rate. Similarly, BDex was found to be very useful for heart involved patients, as it is associated with high cardiac response rate. However, CLD did not show very good efficacy in our analysis. It might have the lowest rates of both hematological and organ response.

As far as we are concerned, this is the first NMA to explore the efficacy of treatments in AL amyloidosis. Since amyloidosis has a low incidence rate, there are few studies on the treatment of this disease and most of them are retrospective. A traditional pairwise meta-analysis performed in 2009 (Mhaskar et al., 2009) pooled one RCT, two prospective studies and nine single-arm studies. It concluded that MDex has similar efficacy to ASCT; however, with more participants enrolled, our NMA found that ASCT could induce better HR and cardiac response than MDex. Another pairwise meta-analysis performed in 2018 (Jiang et al., 2018) found that participants treated with bortezomib combinations have better CR and cardiac response compared with those treated without bortezomib; however, it did not research which kind of bortezomib combinations might be better, our NMA filled this gap by comparing the efficacy of BMDex, BDex, and CyBorD. Besides, our study combined both direct and indirect evidence to confirm the result, and we also performed SUCRA to make the rankings more precise.

As illustrated above, a significant heterogeneity in the comparisons of MDex and ASCT, and a significant difference between direct and indirect evidence for the comparisons of ASCT and BDex were found. According to Dias et al., 2011, one of the measures to avoid inconsistency is to avoid heterogeneity. Furthermore, when we conducted a sensitivity analysis by omitting single study sequentially, we found that the heterogeneity as well as the inconsistency became insignificant after we omitted study “Chihiro 2018” (as shown in **Supplement 7** and **Supplement 8**, respectively). The specific reason might be the too wide a range of the participants’ ages (31~93 years old, as shown in **Table 2**). Interestingly, even if study “Chihiro 2018” was omitted, the efficacy outcomes did not change significantly, which further verified the robustness of our results.

Nonetheless, some limitations of this NMA should be discussed. First, definitions of hematological and organ response have changed from the 10th to the 12th International Symposium on Amyloid and Amyloidosis (Gertz et al., 2005; Comenzo et al., 2012), correspondingly, the enrolled studies adopted various definitions. Therefore, we had to use a series of broad definitions for the efficacy measures. Second, the study could not explore several therapies that had not been investigated in a controlled study: vincristine, doxorubicin, and dexamethasone (VAD), lenalidomide, melphalan, and dexamethasone (L-M-dex), and bortezomib monotherapy, etc. Third, most of the enrolled studies are retrospective, which decreased the grades of the evidence, and this limitation could not be completely made up by performing a design-adjusted analysis. Furthermore, we could not strictly control for the administration time and administration mode due to the limited data, and this might also be the source of some trial heterogeneity. For the same reason, we also failed to make a subgroup analysis. Fourth, there is a rigid limitation to a prospectively defined set of selection criteria which, could be the basis for a minor bias. Fifth, the findings of this study should be supported by exploring the safety of the interventions, but since few studies have reported the corresponding data, we were unable to analyze the safety right now. Sixth, we failed to register this NMA prospectively, which might also cause a certain bias.

## Conclusion

In conclusion, this is the first NMA to compare the HR, CR, renal and cardiac response rates of the therapies for AL amyloidosis. Among the seven treatments, BMDex was recommended as the most efficient one. However, more RCTs are needed to confirm the efficacy of the therapies directly in our study and research whether administration time and administration mode are relevant to the curative efficacy. In addition, the safety and cost of these drugs also remains to be investigated.

## Author Contributions

Study design: YC and SX. Data collection: NL and SL. Data analysis: YC and SX. Writing: YC and GX.

## Funding

This work was supported by the National Natural Science Foundation of China (No.81970583), the Supporting Project for the Foregoers of Main Disciplines of Jiangxi Province (No. 20162BCB22023), the “5511” Innovative Drivers for Talent Teams of Jiangxi Province (No. 20165BCB18018) and the Nature Science Foundation of Jiangxi Province (No. 20181BAB205016).

## Conflict of Interest

The authors declare that the research was conducted in the absence of any commercial or financial relationships that could be construed as a potential conflict of interest.
