# Supplementary material for: Efficacy of Chemotherapies and Stem Cell Transplantation for Systemic AL Amyloidosis: A Network Meta-Analysis
Source: Front Pharmacol. 2020 Jan 28;10:1601. doi: 10.3389/fphar.2019.01601 (PMC6997776; doi:10.3389/fphar.2019.01601)

**Supplement 1.** The selection criteria with a “PICOS” structure for the enrolled studies:

| Items | Specific Criteria |
| --- | --- |
| Patients | Patients were at least 18 years old and had biopsy-proven systemic AL amyloidosis |
| Interventions/Comparisons | Interventions/Comparisons included ASCT, MDex, BMDex, CTD, BDex, CyBorD, CLD |
| Outcomes | Outcomes were haematological response (HR), complete response (CR), renal response or cardiac response |
| Study designs | Studies were randomized controlled trials (RCTs) or observational controlled trials (OCTs) |

ASCT: autologous stem cell transplantation; MDex: Melphalan + dexamethasone; BMDex: bortezomib + melphalan + dexamethasone; CTD: thalidomide + cyclophosphamide + dexamethasone; BDex: bortezomib + dexamethasone; CyBorD: bortezomib + cyclophosphamide + dexamethasone; CLD: cyclophosphamide + lenalidomide + dexamethasone.

**Supplement 2.** The quality of enrolled randomized controlled studies evaluated by version 2 of the Cochrane risk-of-bias tool for randomized trials (RoB 2):

Fig. 1: Risk of bias summary of enrolled RCTs


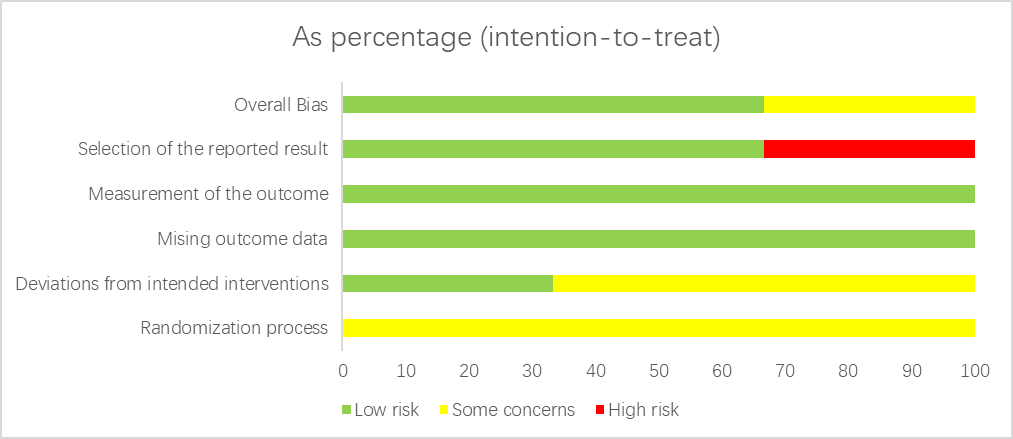


Fig. 2: Risk of bias graph of enrolled RCTs:


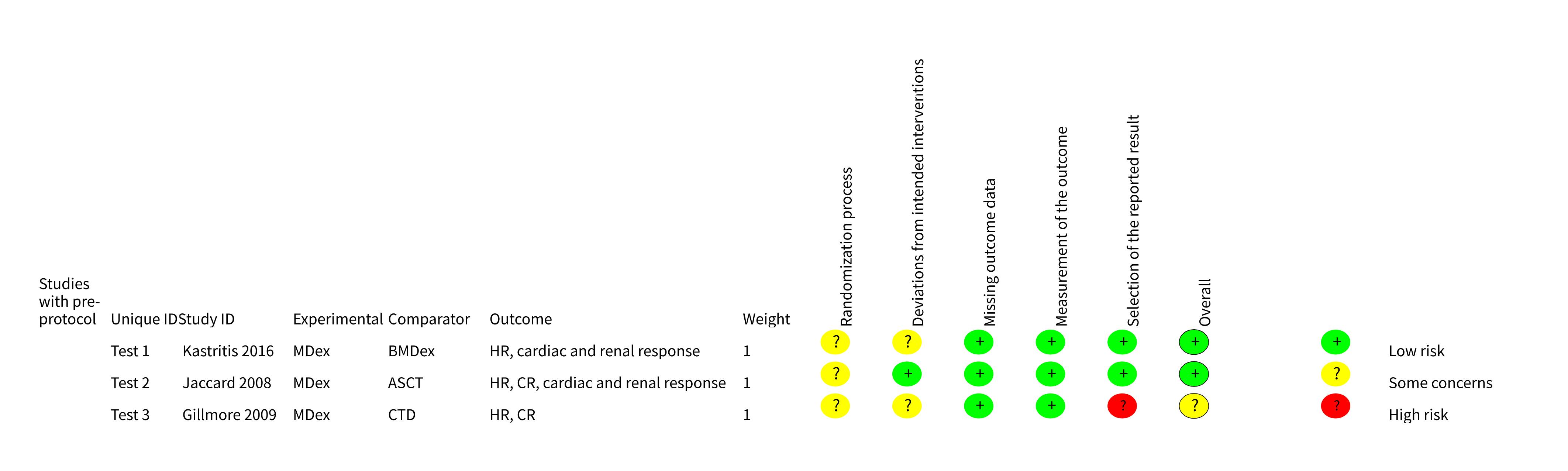


**Supplement 3.** The diagnostic and trace plots for the convergence of this network meta-analysis

Fig. 1 Haematological response


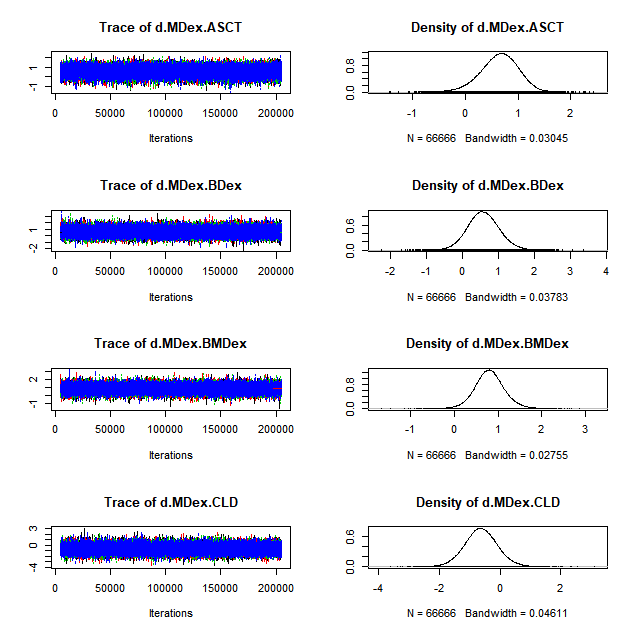


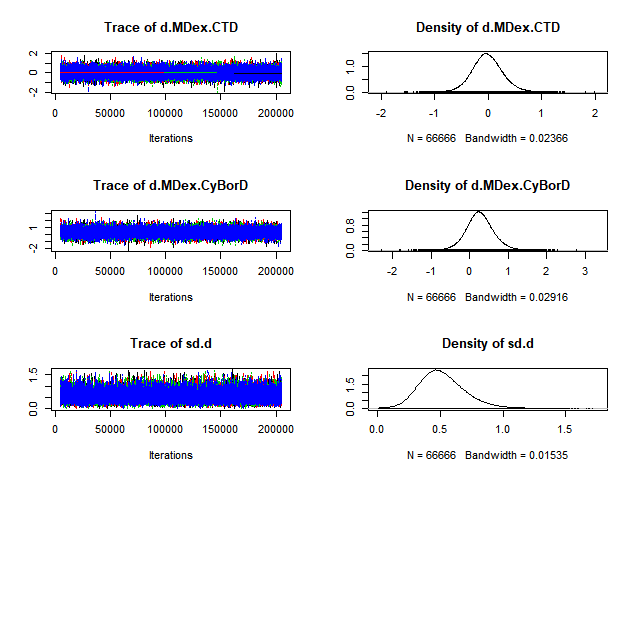


Fig. 2 Complete response


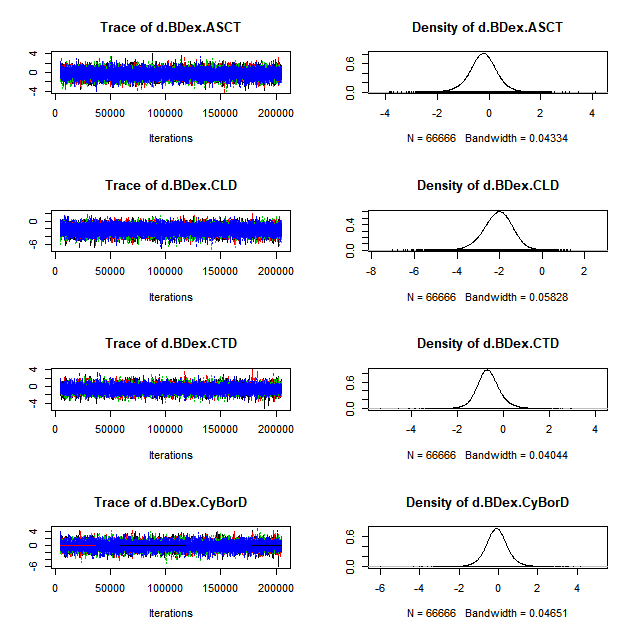


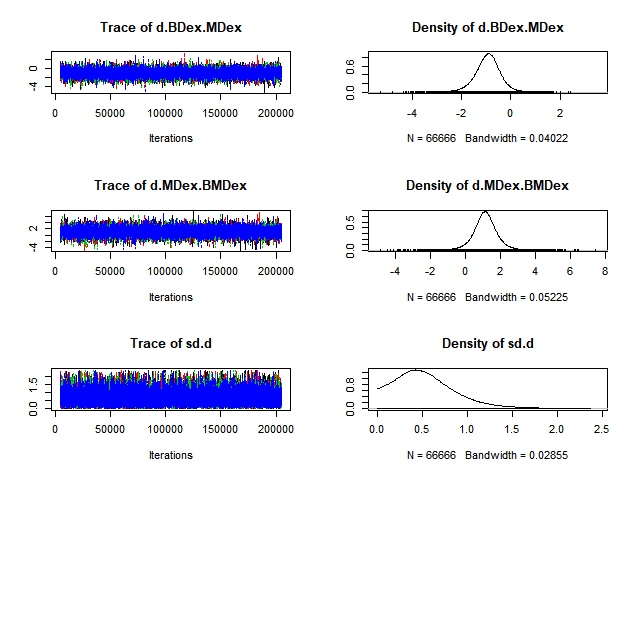


Fig. 3 Renal response


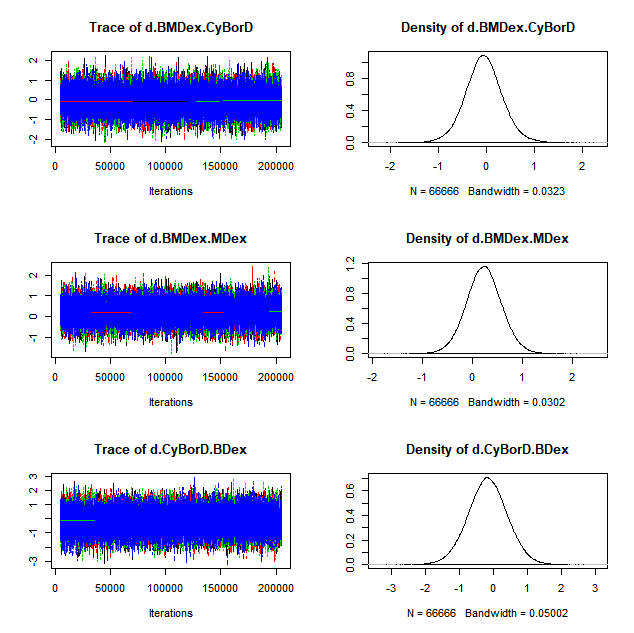


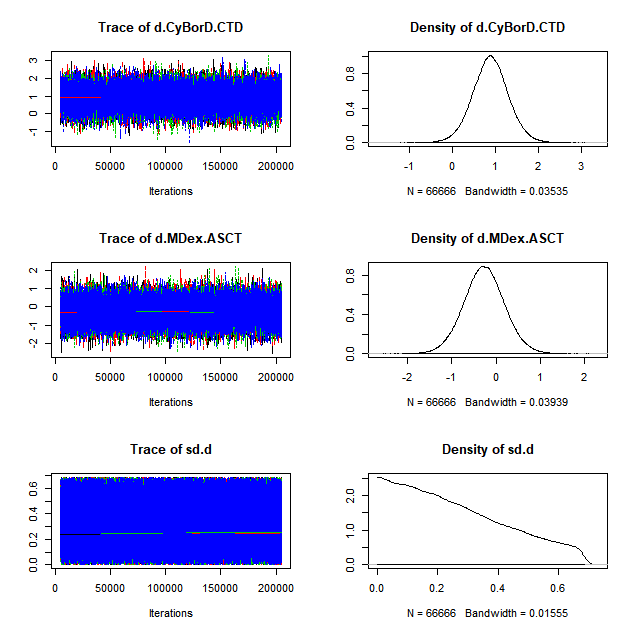


Fig. 4 Cardiac response


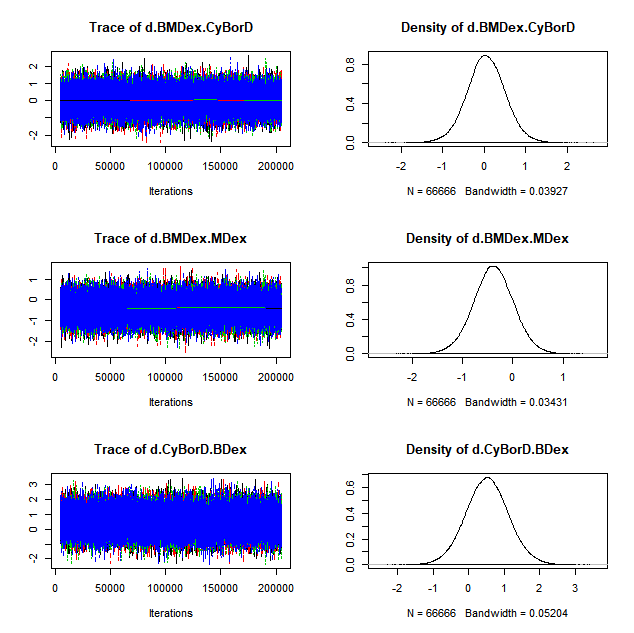


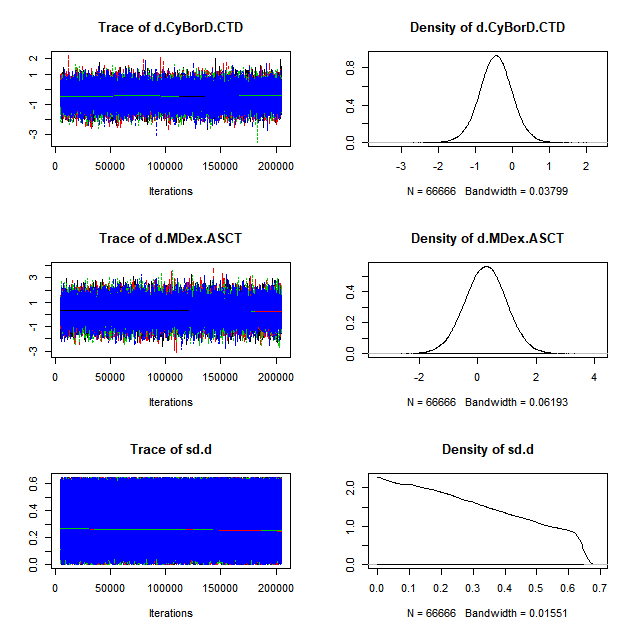


**Supplement 4.** The heterogeneity of both pairwise meta-analysis and network meta-analysis

Fig. 1 Haematological response


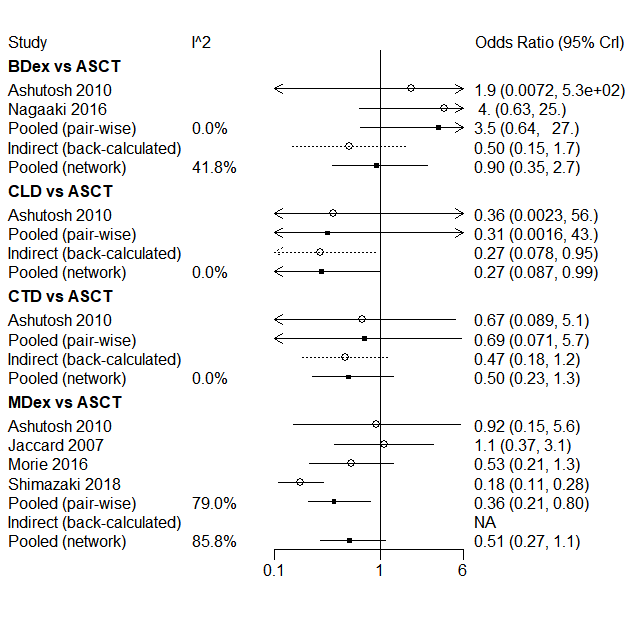


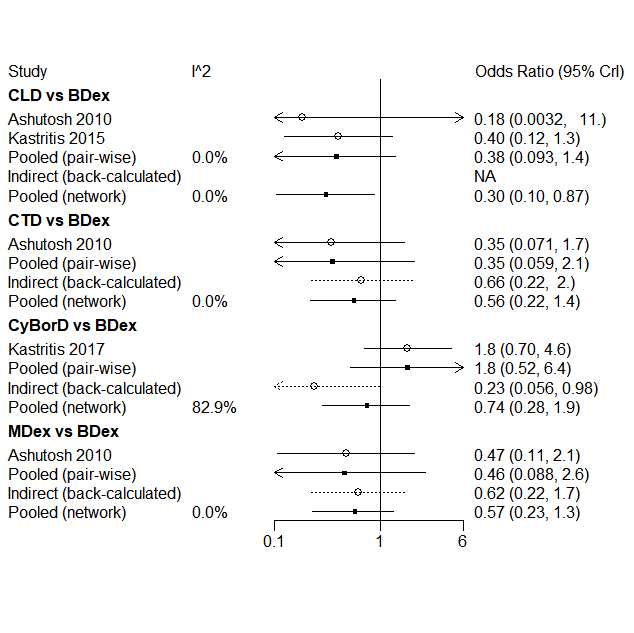


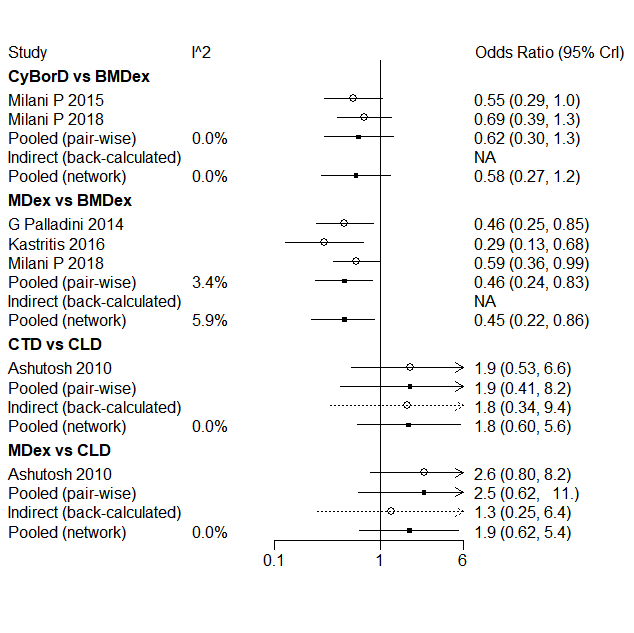


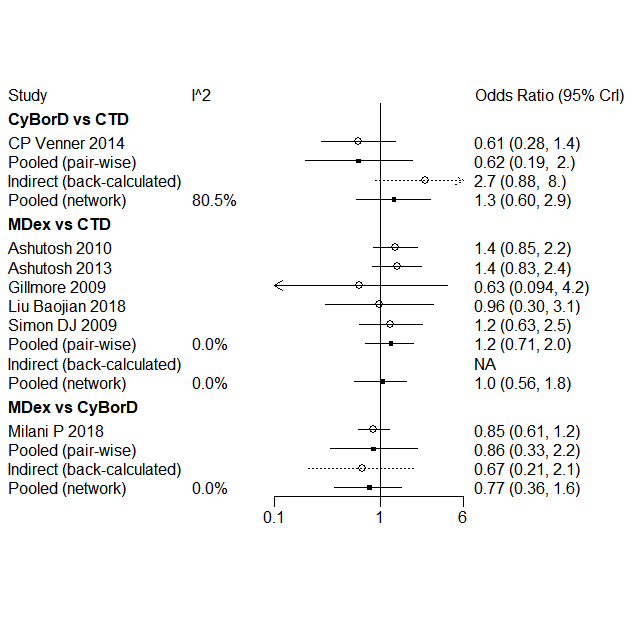


Fig. 2 Complete response


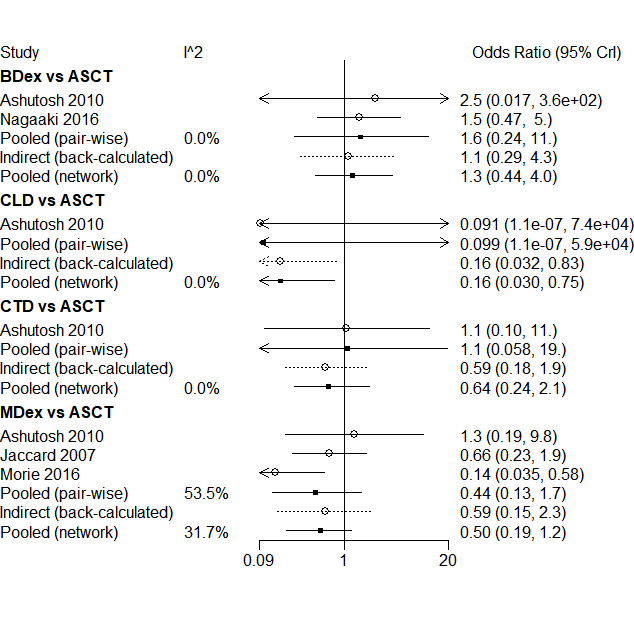


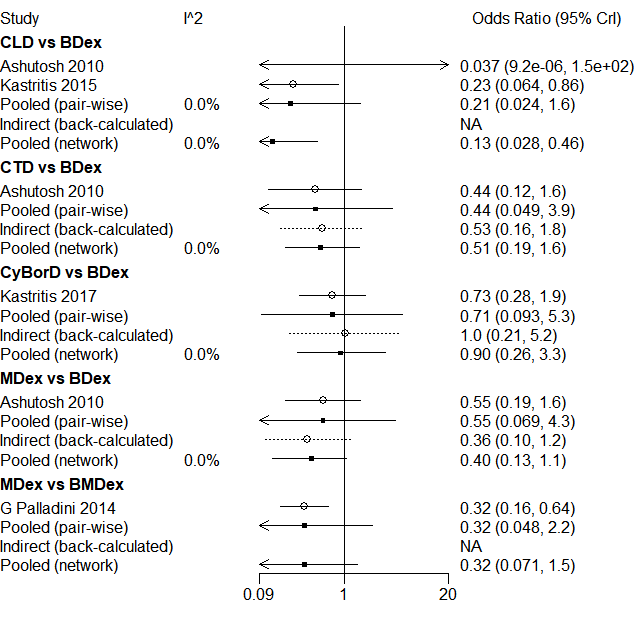


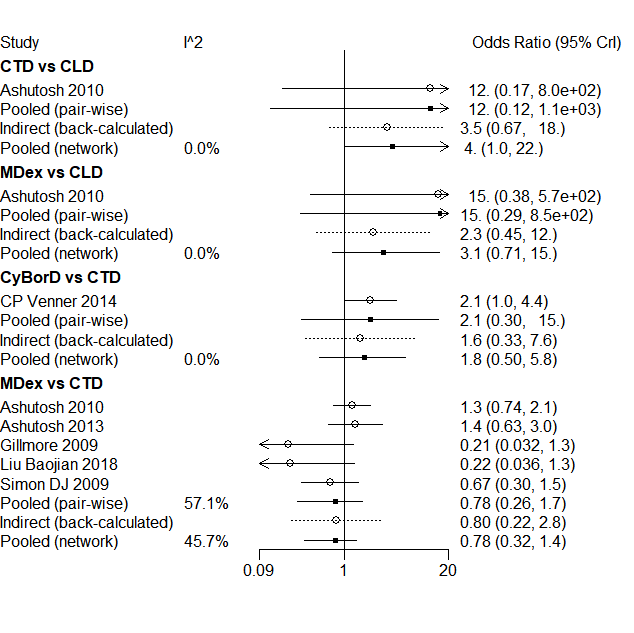


Fig. 3 Renal response


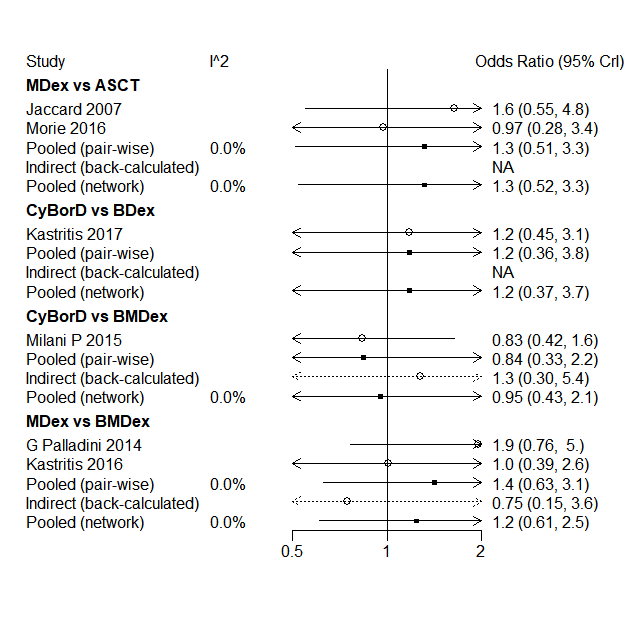


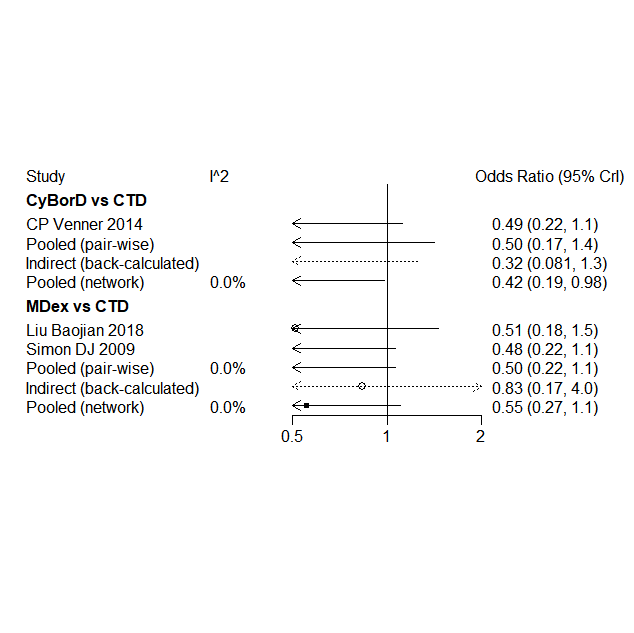


Fig. 4 Cardiac response


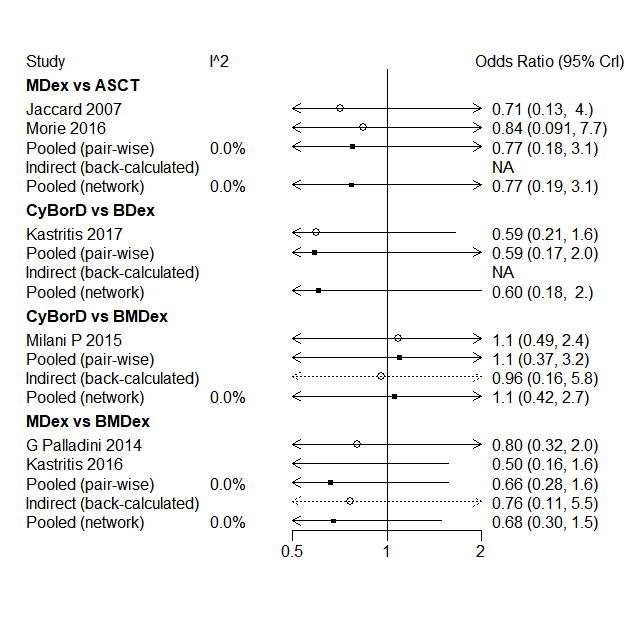


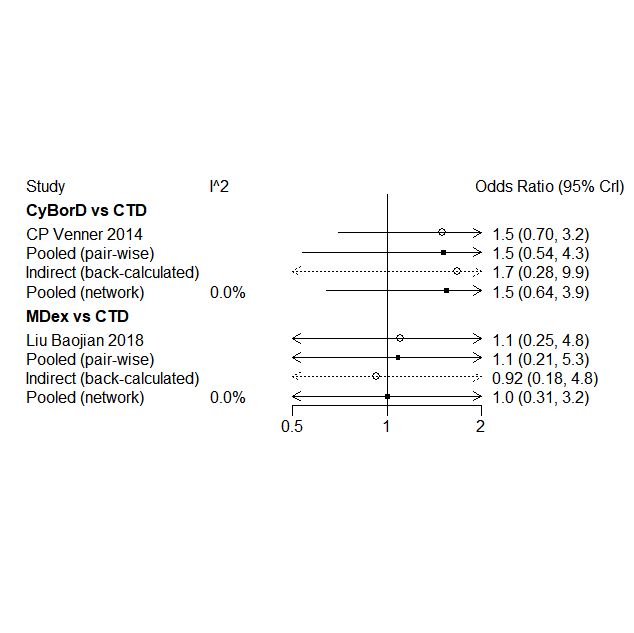


**Supplement 5.** Comparisons between direct and indirect evidence

Fig. 1 Haematological response


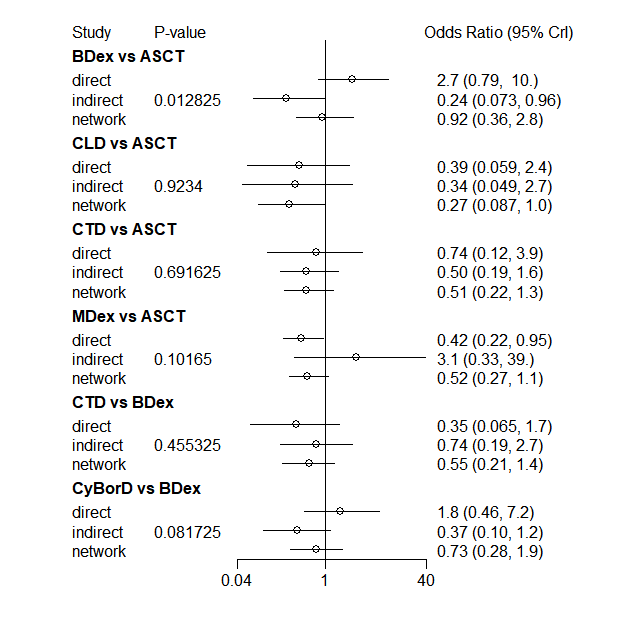


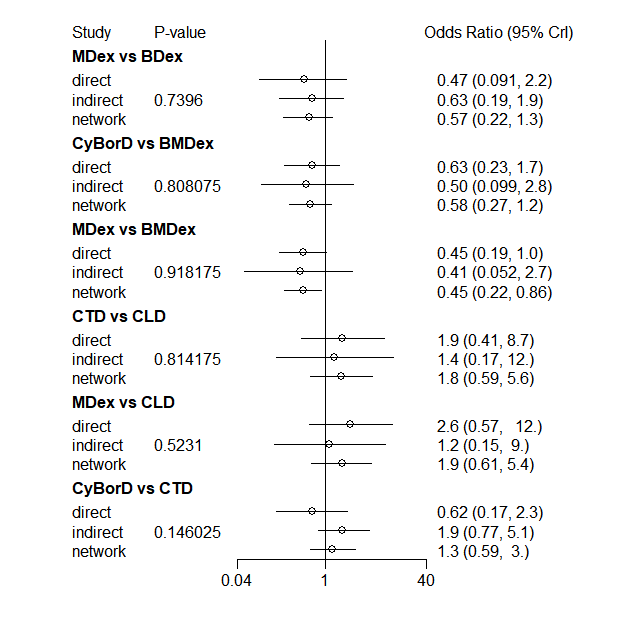


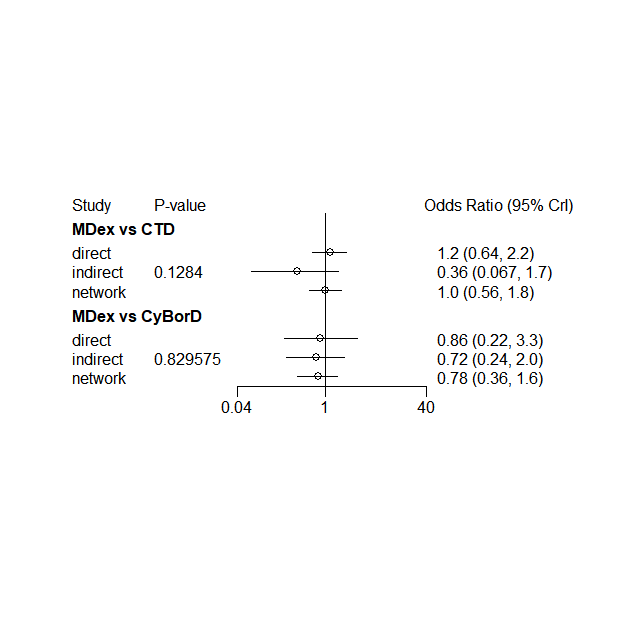


Fig. 2 Complete response


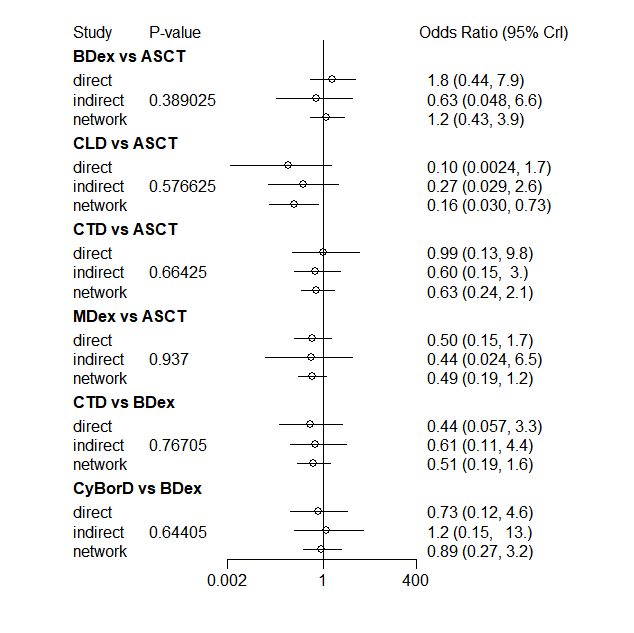


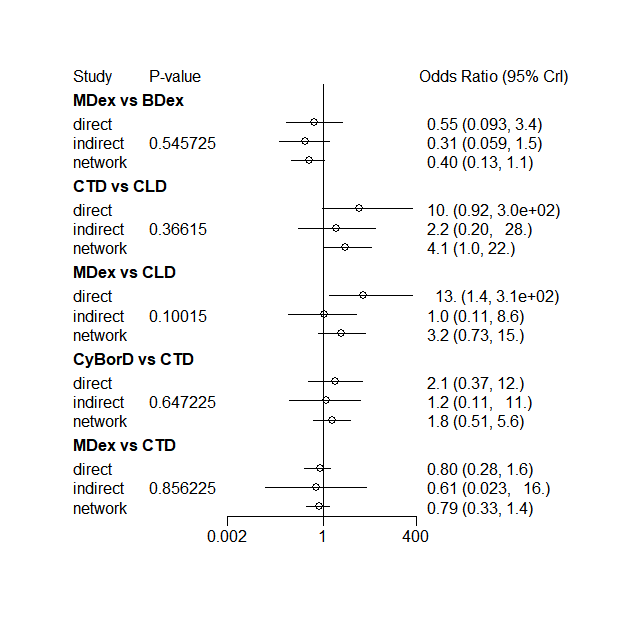


Fig. 3 Renal response


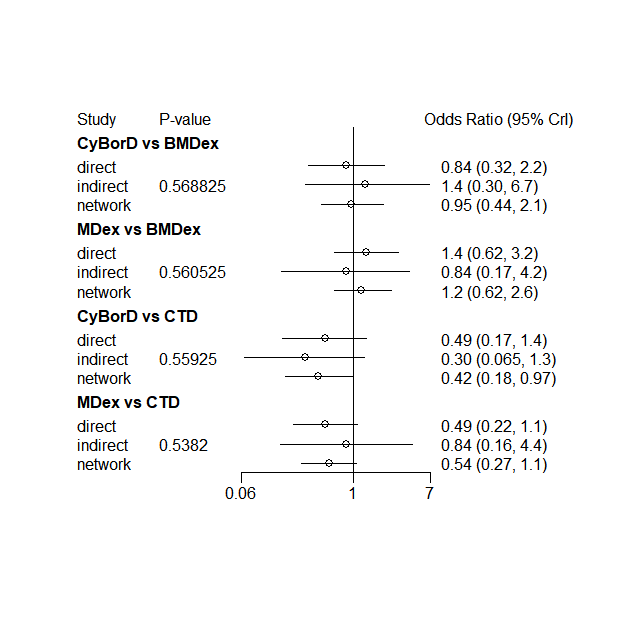


Fig. 4 Cardiac response


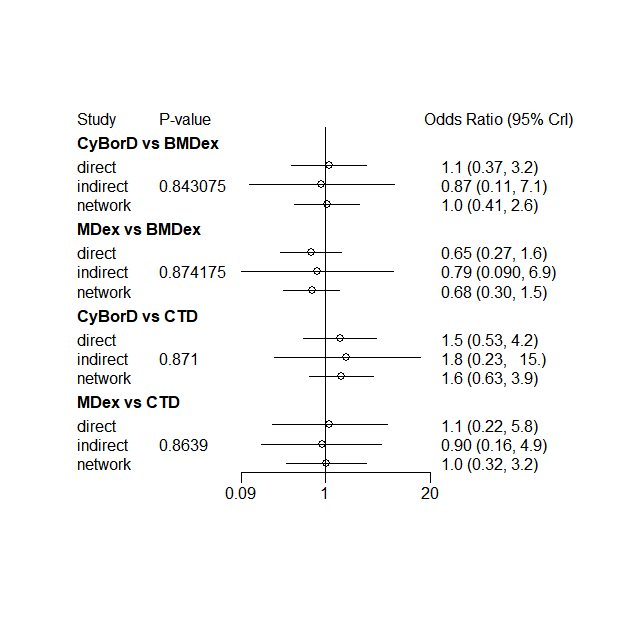


**Supplement 6.** Funnel plots for publication bias

Fig. 1 Haematological response

Fig. 2 Complete response

Fig. 3 Renal response

Fig. 4 Cardiac response

**Supplement 7.** The heterogeneity of both pairwise meta-analysis and network meta-analysis without study “Chihiro 2018”

Fig. 1: The insignificant heterogeneity for HR


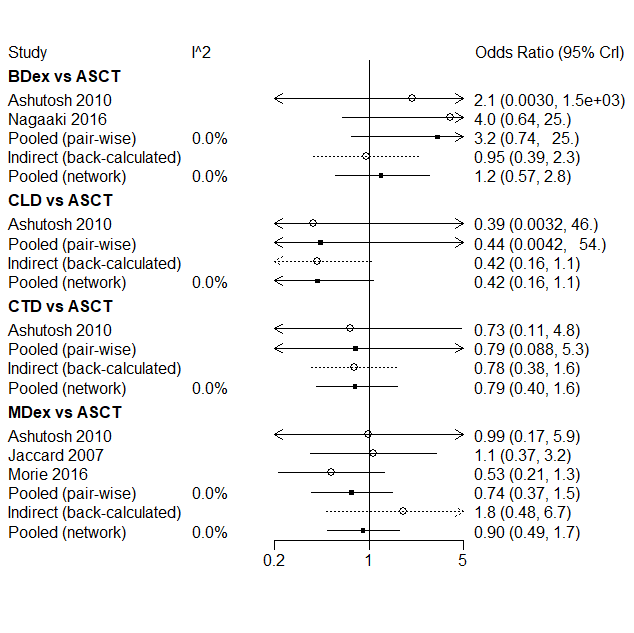


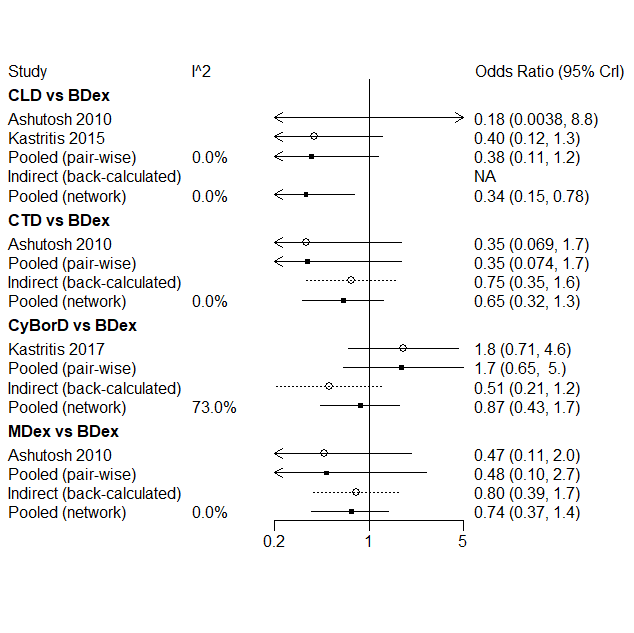


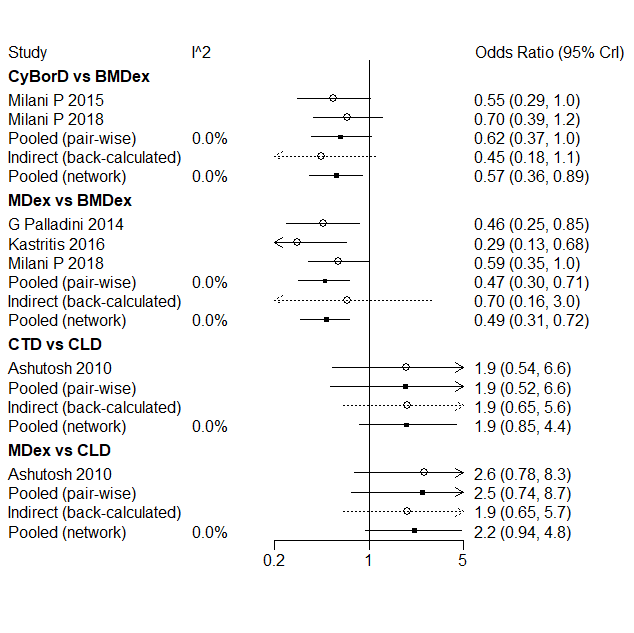


Fig. 2: The insignificant heterogeneity for HR


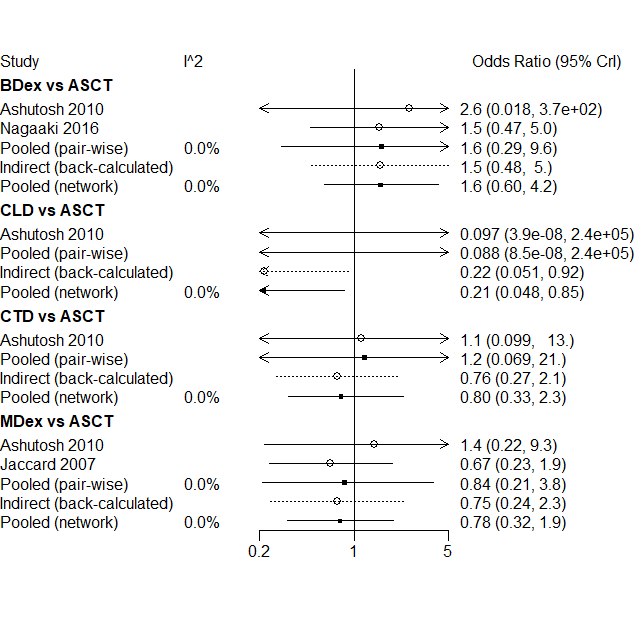


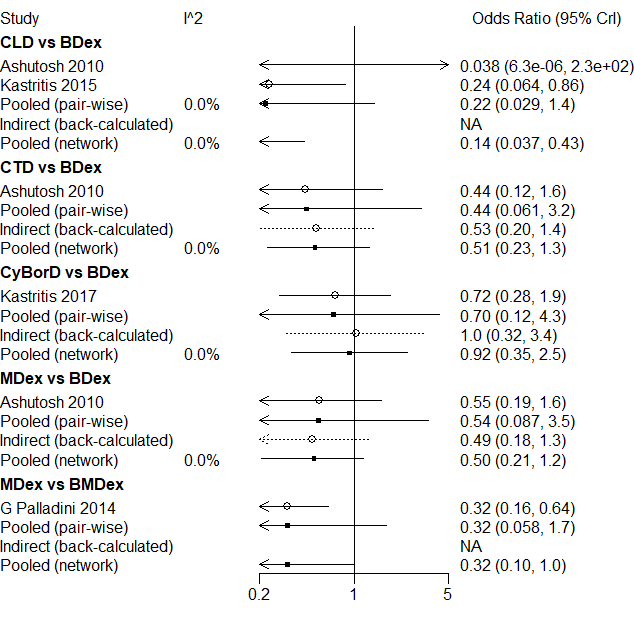


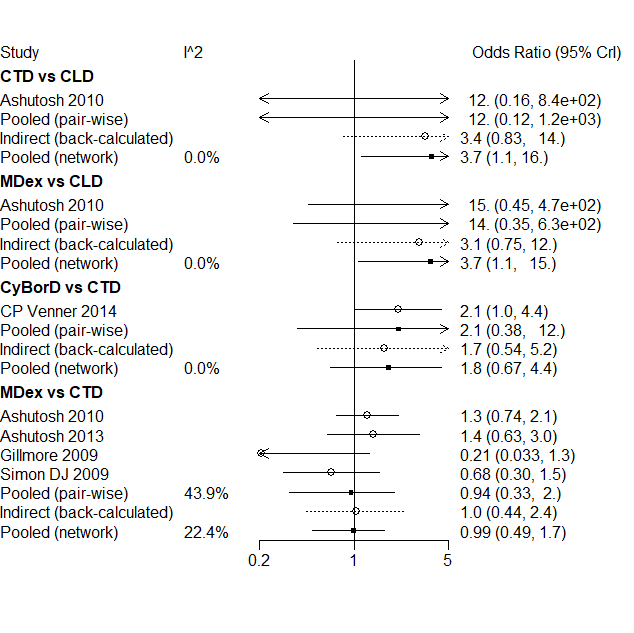


**Supplement 8.** Comparisons between direct and indirect evidence for HR without study “Chihiro 2018”


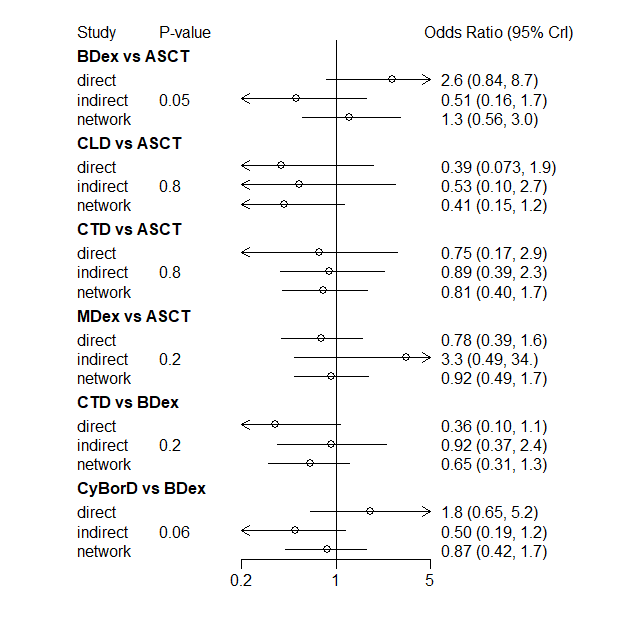


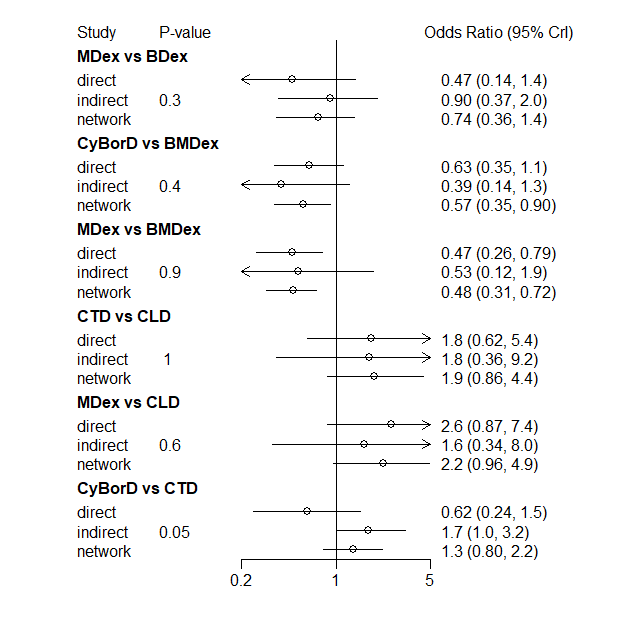


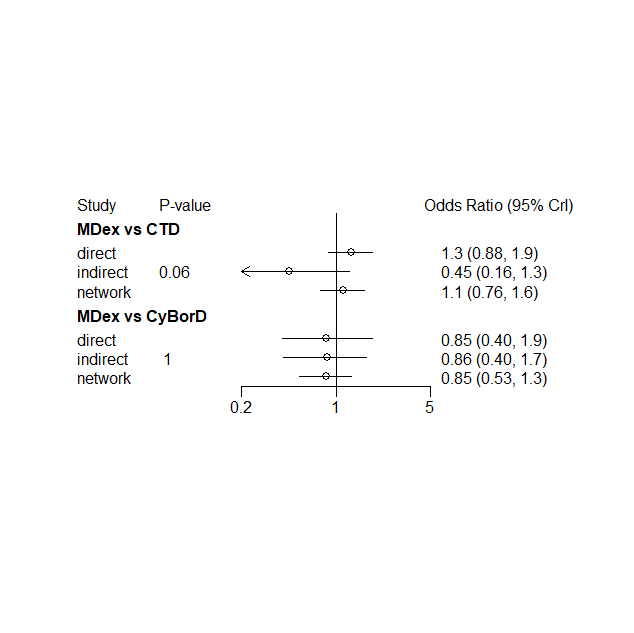

Supplement: Supplementary file 1 [file DataSheet_1.doc]
